# Feasibility of Contrasting Brain Connectivity Patterns in Cognitive and Motor Cerebral Networks to Clinical Outcomes in Patients Surviving Acute Respiratory Failure: A Pilot Study

**DOI:** 10.7759/cureus.17785

**Published:** 2021-09-07

**Authors:** Nathan Morelli, Nathan F Johnson, Evan P Cassity, Anna G Kalema, Peter E Morris, Ashley A Montgomery-Yates, Kirby P Mayer

**Affiliations:** 1 Department of Physical Therapy, High Point University, High Point, USA; 2 Department of Physical Therapy, University of Kentucky, Lexington, USA; 3 Division of Pulmonary, Critical Care and Sleep Medicine, University of Kentucky College of Medicine, Lexington, USA

**Keywords:** post-icu brain connectivity, multifaceted outcomes, neuroimaging biomarkers, cognition, mobility

## Abstract

Background: There is a paucity of research regarding the feasibility and association of cerebral cortex function to patient outcomes after acute respiratory failure (ARF).

Purpose: To determine the feasibility of functional connectivity measures and examine the association of functional connectivity to a multifaceted battery of outcomes in survivors of ARF.

Methods: Eight ARF patients (age:58±3.7, ICU days:10.4±8.6) completed functional magnetic resonance imaging (fMRI), cognitive, physical-function, anxiety, depression, and driving simulator tests at one month post-hospital discharge. Pearson’s correlations assessed the relationship between functional connectivity within the default mode network (FPN), sensorimotor network (SMN), and frontoparietal network (FPN) to outcomes.

Results: Low physical-function (r=0.75, p=0.03) and divided-attention (r=-0.86, p=0.03) during the driving simulator task correlated with low FPN connectivity. Low SMN connectivity demonstrated relationships to slower gait speed (r=0.82, p=0.01) and low short physical performance battery (SPPB) scores (r=0.81, p=0.01).

Conclusions: fMRI is feasible to assess ARF patients’ post-ICU limitations, as low post-ARF brain connectivity may be linked to low physical function, providing potential development of therapeutic interventions.

## Introduction

Patients surviving critical illnesses such as sepsis and acute respiratory failure (ARF) often suffer impairments in cognitive function, decreased mobility, and increased prevalence of psychiatric disorders such as depression and anxiety [1]. Post-intensive care syndrome (PICS) is the collection of “new or worsening problems in physical, cognitive, or mental health status after a critical illness and persisting beyond acute care hospitalization” [2]. One-third of ICU survivors develop significant cognitive and physical function impairments well after hospital discharge [3,4]. Clinical research demonstrates that patient- and ICU-related factors, such as older age and delirium, may increase the risk of long-term impairments [5]. Currently, there is a need to identify underlying pathophysiologic mechanisms of impairments related to PICS that may facilitate the development of intervention strategies.

Despite the paucity of direct or primary neurologic insult, structural and functional alterations to the central nervous system (CNS) have been found in post-ICU survivors, including patients recovering from ARF. Notable post-ICU neuroimaging findings include lesions to white matter, altered brain activity during cognitive tasks, and diffuse grey matter atrophy of the cerebral cortex in those with post-ICU cognitive impairment [6]. The proposed cause of these findings is likely multifactorial in nature, including ICU environment, delirium, arterial blood gas alterations, and systemic metabolic or inflammatory factors [7]. The corresponding findings within critical care survivors of neurophysiologic, functional, and structural abnormalities often mirror changes observed in advanced aging and progressive neurodegenerative disorders, such as Alzheimer’s disease [8]. At this juncture, it remains unclear, how the patterns of cortical functional connectivity relate to post-ICU patients’ outcomes.

To date, neuroimaging has been the primary tool to elucidate mechanisms underlying cognitive deficits in critical illness survivors. However, there is only a limited amount of data concerning post-ICU physical function deficits, and emotional health impairments to functional neuroimaging. The least recognized may be measured crucial to driving performance, such as reaction time. Interestingly, most everyday tasks and societal roles rely on inter-connected regions across the cerebral cortex (i.e., networks) [9]. Despite the known network structure of the cerebral cortex, measures of connectivity after critical illness, particularly ARF, are currently lacking.

Functional connectivity, the amount of signaling coherence in a network, demonstrates significant association to comparable cognitive and motor impairments found in traumatic brain injury, mild cognitive impairment, and Alzheimer’s disease. Briefly, increased connectivity in response to injury or disease is viewed as a compensatory measure and is considered beneficial to maintaining functional performance. Jimenez-Martin et al. [10] were the first to extend resting-state analyses to critical illness survivors of multi-organ failure assessed at six-month post-ICU. Not only was connectivity higher than age-matched controls and correlated with higher severity of organ failure, but post-ICU connectivity was also significantly associated with neuropsychological outcomes. Evidence of connectivity changes after organ failure provides preliminary evidence for altered cortical network function in critical illness survivors and highlights the potential role connectivity assessments may play as a mechanistic biomarker of post-ICU impairments.

The purpose of this study was to determine the feasibility of studying fMRI in ICU survivors along with administration of standardized neurocognitive tools so that the association, in eventual larger studies, may be studied between post-ICU functional connectivity patterns (high or low) and physical function, cognition, emotional health, and driving task reaction time. We hypothesized that greater connectivity within the motor and cognitive networks will be associated with improved physical function, cognition, emotional health, and reaction time post-ICU.

## Materials and methods

Ethical considerations, design, and participants

The study was approved by the internal review board at the University of Kentucky (UK) (MEDFL IRB #44143). Patients provided informed written consent before participating in the study.

STROBE: This study was reported in accordance with the Strengthening the Reporting of Observational Studies in Epidemiology (STROBE) guidelines.

Study design: A prospective, cross-sectional, observational study was conducted with adult patients. Patients were recruited for enrollment at their first follow-up appointment in the UK ICU Recovery Clinic. The study aimed to complete study procedures approximately one month after hospital or last institutional discharge.

Participants: Patients were eligible if they were over 18 years old and had recently been discharged from the hospital after ICU admission for ARF. Participants were excluded if they at the time of enrollment were unable to stand without an assistive device, unable to walk, had an acute stroke, had a concomitant neuromuscular or neurodegenerative disease that could influence cognition or motor performance, and had a current fracture which limited lower extremity weight-bearing or bilateral upper extremity function. To ensure participant safety with MRI scanning, participants were excluded if they were pregnant or voiced suspicions of being pregnant, claustrophobic, possessed metal fragments or medical implants which were non-removable and unsafe for entering a 3-Tesla MRI, or were unable to lay supine for >30-min.

Demographic and clinical variables: Demographics and clinical variables were extracted from Electronic Health Record for participants. Independent variables of interest include age, sex, body-mass index, comorbidity status (Charlson Comorbidity Index), ICU receipt of sedative medication during hospitalization (yes or no), the occurrence of delirium as noted in objective nursing reports (yes or no), the mean and the highest sedative dose and mean and worst agitation status during first 72 hours of ICU admission quantified by the Richmond Agitation-Sedation Scale (RASS) [11], the severity of illness measured by Sequential Organ Failure Assessment scores (SOFA) [12] and Acute Physiology and Chronic Health Evaluation (APACHE-II) [13], number of days requiring mechanical ventilation, peak fraction of inspired oxygen, peak positive end-expiratory pressure, length of ICU and hospital stay, and discharge destination. Physiological variables were extracted based on the clinically worst recorded values in the first 72 hours of admission to the ICU and included arterial partial pressure of oxygen and carbon dioxide, hematocrit, glomerular filtration rate, lactic acid, and creatinine.

Cognitive, physical, and emotional health outcomes

Patients completed a battery of outcome measures as established expert consensus [2] and recommendations [14].

Montreal Cognitive Assessment (MoCA) is a cognitive assessment performed by a trained researcher. The cut-off score of 26 has been shown to demonstrate 90% sensitivity in identifying mild cognitive impairments in older adults. A cut-off score of 23 has also been proposed and appears to lower the false positive rate and improve diagnostic accuracy [15]. The MoCA is comprised of 16 items and 11 categories including visuospatial and executive functions, naming, memory, attention, language, abstraction, and orientation. Higher scores are representative of better global cognitive performance [15].

Hospital Anxiety and Depression Scale (HADS) has been routinely used in a variety of clinical populations, including post-ICU populations, to quantify anxiety and depression [16]. The scale has 14 questions for a total of 42 points and subcategories for anxiety and depression. Higher scores indicate a greater level of anxiety and depression [17].

Impact of Events Scale-Revised (IES-R) is a 22-item self-report questionnaire about distress that captures a provisional diagnosis of post-traumatic stress disorder (PTSD) with a score >33/88 [18].

EuroQol-5Dimension-5L (EQ-5D) questionnaire contains a visual analog scale (VAS) and five questions of five domains, including mobility, self-care, usual activities, pain/discomfort, anxiety/depression, which provides a descriptive profile of overall health status. This measure has been used previously to quantify the quality of life outcomes in the critical illness of respiratory origin [19].

Short physical performance battery (SPPB) is a physical function assessment comprising balance, four-meter gait speed, and sit-to-stand tasks. Each task component is scored on a scale of zero to four, with a sum score of 12. Lower scores are associated with mobility deficits and negative outcomes in older patients after hospital discharge [20]. The SPPB was administered by a licensed physical therapist.

Driving simulator assessed driving-related reaction time during an eight-minute simulated divided attention drive. The driving simulator was equipped with STISIM® Drive technology. (STISIM Drive, Systems Technology Inc., Hawthorne, CA). The simulator is outfitted with a 135° horizontal field of view that covers three integrated screens along with a functional steering wheel, turn signals, throttle, and brake. The system is also equipped with high-speed graphics, naturalistic driving sounds such as engine and external road noises during simulations, and a simulated dashboard with a speedometer. The software is interactive to the participant’s steering, acceleration, and braking and provides continuous data collection. During the simulated drive, participants were required to maintain lane position and speed while attending to changing stimuli on the computer screen. Briefly, diamond-shaped icons were presented on the upper left and right sides of the simulated windshield. One of the two diamond-shaped icons would change to a right-facing arrow or a left-facing arrow during the simulated drive. Participants were instructed to press one of two red buttons on the console once they noticed a change. A right button press corresponded to a right-facing arrow and a left button press corresponded to a left-facing arrow. Participants were instructed to attend to the direction of the arrow and not the side of the monitor, left or right, the stimuli were presented on. Inter-stimulus intervals corresponded to distance traveled, so the speed at which each stimulus changed was dependent on the speed at which the participant was driving. Participants were instructed to maintain a speed of 35 miles per hour. Reaction time and accuracy were recorded for 48 total trials.

MRI data acquisition: Structural and functional MRI was collected using a 3.0 T TIM Siemens scanner and completed the same day as all behavioral testing. High-resolution structural images were collected in a magnetization prepared rapid gradient echo (MPRAGE) three-dimensional T1-weighted sequence with the following parameters: Repetition Time (TR) = 2,530 ms, Echo Time (TE) = 2.3 ms, Field of View = 256 mm x 256 mm. Resting-state functional MRI (fMRI) scans were 10 min in duration during which, participants were instructed to keep their eyes open. Resting-state functional data was collected using the following parameters: TR = 607 ms, TE = 32 ms, Field of View = 1,760 mm x 1,760 mm.

MRI data processing: T2*-weighted images were preprocessed using FSL (version 6.1) (FMRIB Software Library; https://fsl.fmrib.ox.ac.uk). Functional brain volumes from all subjects underwent slice timing and head motion correction, non-brain removal using BET, and high pass temporal filtering. Additionally, data underwent spatial smoothing using a Gaussian kernel of full-width at half-maximum of 5 mm resolution. Functional images were then coregistered onto the individual’s high-resolution T1-weighted images and then normalized to standard space (Montreal Neurological Institute atlas, using resolutions of 2 x 2 x 2 mm) using combined affine and nonlinear registration (FSL FNIRT, with warp resolution = 10 mm).

**Figure 1:**
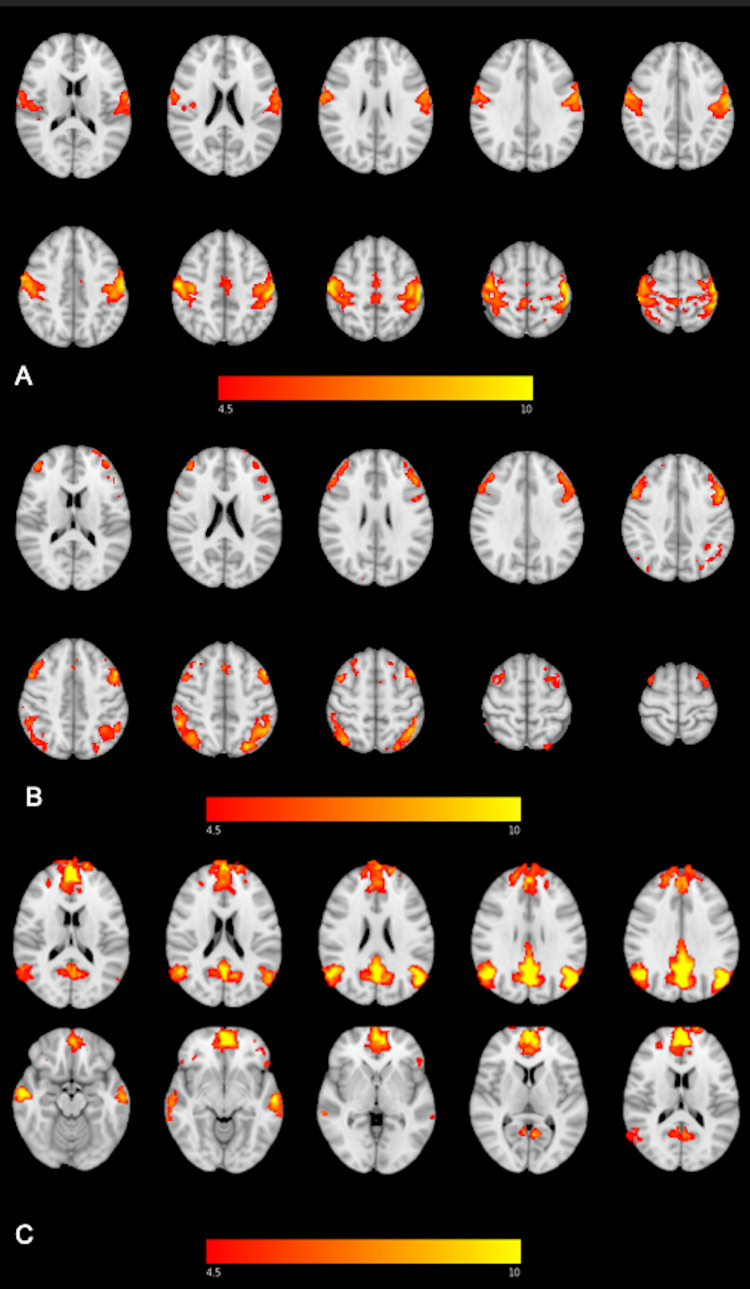
Resting-state functional connectivity maps of the (A) sensorimotor network (SMN), (B) frontoparietal network (FPN), and (C) default mode network (DMN).

Each participant’s normalized fcMRI data were concatenated across time to form a single 4D image and underwent single-subject independent component analysis (ICA) using FSL Multivariate Exploratory Linear Optimized Decomposition into Independent Components (MELODIC). ICA is a data-driven approach to separate multivariate data into statistically and spatially independent components. For application to the individual participant’s functional connectivity data, ICA generates components of 4D signal and noise. Noise within each patient’s 4D data was identified using AROMA and removed using the fsl_regfilt command. Cleaned functional data for all participants who underwent group-level ICA to generate 20 independent components through MELODIC. Components representing the default mode network (DMN), sensorimotor network (SMN), and frontoparietal network (FPN) were identified in standard space and are depicted in Figures 1A-1C. These networks are associated with executive function and visuospatial processing (i.e., FPN) [21], introspective cognitive functions (i.e., DMN), and sensorimotor control (i.e., SMN) [18]. Network masks were created using 4 mm spheres of significant clusters or regions of interest (ROIs) within each network and transferred into each subject’s native space using FLIRT. MNI coordinates are outlined in Table 1. Connectivity measures from all networks underwent Fisher’s r-to-z transformation and were averaged across intranetwork ROIs.

**Table 1:** MNI coordinates for all regions of interest incorporated into the DMN, FPN, and SMN networks. DMN - Default Mode Network, FPN - Frontoparietal Network, SMN - Sensorimotor Network

	X	Y	Z
DMN			
Left Lateral Occipital Lobe	70	31	52
Right Lateral Occipital Lobe	22	32	51
Right Ventromedial Prefrontal Cortex	49	91	32
Precuneus	45	29	52
FPN			
Left Lateral Parietal Lobe	58	26	60
Left Middle Frontal Gyrus	70	70	58
Right Lateral Parietal Lobe	25	31	62
Right Middle Frontal Gyrus	23	68	64
SMN			
Left Postcentral Gyrus	70	48	65
Right Precentral Gyrus	43	54	66
Right Postcentral Gyrus	21	53	66

Statistical analysis

 Descriptive statistics were summarized using median and interquartile ranges. Shapiro-Wilks test was used to determine data normality. The relationship between outcome measures and functional connectivity within the DMN, SMN, and FPN were assessed using Pearson’s correlation coefficients. Correlations were interpreted as little to no relationship (0-0.25), fair (0.26-0.5), moderate to good (0.51-0.75), and strong (>0.75) [22]. Significance was set a-priori at p≤0.10 due to the small sample size [23]. All statistics were completed in SPSS version 25 (IBM, Armonk, NH).

## Results

Eleven patients agreed to enroll in this study. Eight of the 11 (73%) patients were able to complete fMRI testing. Two patients were unable to tolerate the fMRI equipment with complaints of increased anxiety and the other patient was deemed ineligible on safety review due to an indwelling central port. The eight patients completing the study had a median age of 58.0[56.3-60.8] years, 50% were male, APACHE 23.0[13.8-26.0], and SOFA score of at ICU 10.5[5.3-11.8]. Detailed demographic and clinical variables are presented in Table 2. Patients’ comorbidities and admitting diagnoses are outlined in Table 3.

**Table 2:** Demographic and clinical information. Abbreviations: Y - yes, N - no, BMI - body mass index, CCI - Charlson Comorbidity Index, RASS - Richmond Agitation-Sedation Scale, SOFA - Sequential Organ Failure Assessment, APACHE - Acute Physiology and Chronic Health Evaluation, ETT - endotracheal tube, HFNC - high-flow nasal cannula, FIO_2_ - fraction of inspired oxygen, PEEP - positive end-expiratory pressure, GFR - glomerular filtration rate, LOS - length of stay, ICU - intensive care unit. Median [Interquartile Range]

Variable	ICU (Median [Interquartile Range])
Age	58.0[56.3-60.8]
BMI	33.7[30.5-36.8]
Sex	4 Females, 4 Males
Sedation (Y/N)	6 Y
Delirium (Y/N)	4 Y
CCI	3.0[3.0-5.0]
RASS Peak	-4.5[-5.0- -1.25]
RASS Mean	-1.7[-2.7- -0.6]
SOFA	10.5[5.3-11.8]
APACHE	23.0[13.8-26.0]
Ventilation Days	4.1[3.3-11.5]
Oxygen Delivery Mode	6 (ETT), 4 (HFNC)
FIO2 Peak	70.0[52.5-100.0]
PEEP Peak	8.5[3.1-15.0]
PaO2	69.0[57.5-78.8]
PCO2	50.0[31.8-63.8]
Acute Kidney Injury (Y/N)	4 Y
GFR	40.0[24.0-52.8]
Lactic Acid (arterial)	1.8[1.1-2.9]
Creatinine	1.4[1.1-2.7]
ICU LOS	6.5[5.0-14.5]
Hospital LOS	11.5[7.5-19.8]
Discharge Destination	4 (Home), 4 (Acute Rehabilitation)

**Table 3:** Pre-existing conditions, comorbidities, and diagnoses of participants. Abbreviations: PTSD - post-traumatic stress disorder, HTN - hypertension, HLD - hyperlipidemia, DM - diabetes mellitus, COPD - chronic obstructive pulmonary disorder, OSA - obstructive sleep apnea, CKD - chronic kidney disease, CAD - coronary artery disease, A-fib - atrial fibrillation, HFrEF - heart failure with reduced ejection fraction, PVD - peripheral vascular disease, ARF - acute respiratory failure, AMS - altered mental status, COVID - coronavirus disease

Participant #	Pre-existing mental illness	Comorbidities	Diagnosis
1		HTN	ARF, Sepsis, Acute Encephalopathy
2		HLD, HTN, DM, COPD, OSA	ARF, Pneumonia
3	Depression and Anxiety	HTN, OSA	ARF, Hypertensive emergency with Pulmonary Edema
4		HTN, COPD, asthma	ARF, Flu Positive, COPD exacerbation
5		HLD, HTN, CKD, CAD, A-fib, HFrEF	ARF, Flu Positive,
6		HLD, DM, CAD, CKD	ARF, AMS, Pneumonia
7	Depression	HLD, HTN	ARF, COVID
8	Anxiety	HTN, DM, PVD	ARF, COVID, Sepsis

Multiple physiological variables demonstrated moderate correlation to low brain connectivity; however, none reached significance. Low DMN connectivity expressed moderate correlation to mean RASS scores within the first 72 hours of admission (r=-0.56, p=0.15), mechanical ventilation days (r=0.57, p=0.14), Glomerular Filtration Rate (GFR) (r=0.62, p=0.10), and creatinine (p=-0.66, 0.07). Low SMN connectivity was moderately correlated to the number of days on ventilation (r=0.56, p=0.15) and low FPN connectivity demonstrated moderate correlation to peak PEEP (r=-0.56, p=0.15). Additionally, low DMN connectivity was moderately correlated with ICU (r=0.61, p=0.10) and hospital (r=0.62, p=0.10) length of stay.

Cognitive, physical, and emotional health

Five patients (62%) and two patients (25%) met the criteria for mild cognitive impairment for two established cut points, <26/30 and <23/30 respectively on MoCA. Three patients met the criteria for a provisional diagnosis of PTSD according to self-report IES-R. Six (75%) of patients met the criteria for anxiety (8) and three (37%) of patients for depression (8) on respective subscales of HADS [16]. Four (50%) patients had a physical dysfunction defined as less than 8 on the SPPB [24]. Descriptive statistics for clinical measures of cognition, anxiety, depression, and physical function are presented in Table 4.

**Table 4:** Descriptive statistics for clinical outcome measures. Abbreviations: MoCA - Montreal Cognitive Assessment, SPPB - Short Physical Performance Battery, HADS - Hospital Anxiety and Depression Scale, EQ-5D - EuroQol-5 Dimension, IES-R - Impact of Events Scale-Revised. Median [Interquartile Range]

Measure	Post-ICU Performance
MoCA	24.5[21.0-25.8]
EQ-5D	67.5[52.5-75.0]
IES-R	28.0[10.5-53.5]
HADS-Depression	6.5[4.3-10.0]
HADS-Anxiety	10.0[7.3-11.8]
HADS-Total	17.0[12.0-21.5]
Gait Speed (m/s)	0.8[0.5-0.9]
5x Sit-to-Stand	16.8[10.3-26.9]
SPPB-Total	8.0[4.0-10.75]
Driving Divided Attention(s)	1.7[1.4-1.9]

Post-ICU connectivity demonstrated fair to strong correlation with multiple clinical outcome measures (see Table 5). The DMN connectivity demonstrated moderate correlations with MoCA scores (r=0.51, p=0.19), HADS - Depression subscale (r=0.52, p=0.18), and gait speed (0.56, p=0.15). FPN connectivity demonstrated fair to moderate correlation with HADS - Anxiety subscale (r=-0.61, p=0.11), gait speed (r=0.54, p=0.17), 5x sit-to-stand performance (r=-0.50, p=0.20). Physical function (SPPB) (r=0.75, p=0.03) and divided-attention (r=-0.86, p=0.03) during the driving simulator task demonstrated strong correlation to FPN connectivity. SMN connectivity demonstrated significant association to gait speed (r=0.82, p=0.01) and SPPB-Total (r=0.81, p=0.01), as well as moderate correlation to 5x sit-to-stand performance (r=-0.57, p=0.14).

**Table 5:** Correlations between frontoparietal network (FPN), default mode network (DMN), and sensorimotor network (SMN) connectivity to clinical outcomes. Abbreviations: MoCA - Montreal Cognitive Assessment, SPPB - Short Physical Performance Battery, HADS - Hospital Anxiety and Depression Scale, EQ-5D - EuroQol-5 Dimension, IES-R - Impact of Events Scale-Revised. *= p<0.1

	FPN	DMN	SMN
MoCA	-0.29	0.51	-0.01
EQ-5D	0.28	0.48	0.47
HADS-Depression	-0.43	0.52	0.18
HADS-Anxiety	-0.61	0.18	-0.17
HADS-Total	-0.54	0.37	0.00
IES-R	-0.23	-0.07	0.01
Gait speed	0.54	0.56	0.82*
5x sit-to-stand	-0.50	-0.32	-0.57
SPPB-Total	0.75*	0.30	0.81*
Driving - Divided Attention Reaction Time	-0.86*	0.00	-0.47

## Discussion

The findings of our pilot study demonstrate that measures of resting-state connectivity are feasible in patients who survive an ICU stay for ARF and may be contrasted to cognitive, physical, and emotional status testing within the early outpatient setting. Moreover, these data suggest low post-ICU functional cortical connectivity is related to slower gait speed, worse physical function (SPPB), and poor performance on divided-attention tasks within a driving simulation. Specifically, lower (i.e., worse) FPN and SMN connectivity were statistically related to lower physical function. Thus, we demonstrate that cortical network connectivity networks may play a major role in physical recovery for patients surviving ARF. The alterations in cortical networks, specifically lower connectivity, maybe a clinically relevant pathologic mechanism leading to worse outcomes such as poor dual-task performance. In addition, the data may suggest that cortical changes in the DMN, FPN, and SMN are significantly related to cognitive and emotional health impairments; however, the small sample and exploratory nature of the study may limit the strength of these arguments.

The present findings suggest that greater disease severity, sedation, and agitation, and decreased renal function in patients surviving ARF may be associated with greater connectivity in networks associated with cognitive and motor functions. Perhaps acting as an acute compensatory response, increased connectivity has been previously associated with worse short and long-term outcomes in aging and neurodegenerative disease [25]. Prior reports of increased connectivity after neurologic disease and traumatic brain injury support this hypothesis [25]. Therefore, the findings of our study may provide a foundation for further investigation to determine whether acute critical illness and ICU treatments may portend structural and functional changes in cortical networks.

To our knowledge, this is the first study in patients surviving the medical ICU to assess reaction times and performance during a driving simulation after critical illness. Driving is a complex task that is essential to occupational responsibility and activity of daily living, but it also requires rapid cognitive processing (i.e., visuospatial processing, attention, task-switching) and motor responses to react to environmental stimuli [26]. Furthermore, return to work is frequently assessed after critical illness [26], but return to driving and driving performance is rarely addressed. Driving is an important instrumental activity of daily living (iADL) that may signify independence and a major milestone in recovery. Future investigations with larger sample sizes are needed to adequately determine how critical illness influences this salient skill.

Post-ICU connectivity within networks serving sensorimotor system, attention, cognitive control, self-referential thought, and episodic memory [27] demonstrated moderate to strong relationships to cognition, driving divided-attention reaction time, and physical function. Cognitive deficits are well documented in critical illness survivors and have been associated with structural and task-based functional MRI findings [28]. While the average performance on the MoCA did not fall below the conservative cut-off score [15], DMN connectivity demonstrated a positive association with overall cognitive function in this cohort. This finding is consistent with the association of DMN connectivity to post-ICU cognitive function at three-month follow-up seen in patients surviving multi-organ failure [10]. Taken together, these findings highlight the possible role of DMN connectivity as a biomarker of cognitive function in critical illness survivors.

Additionally, connectivity after ARF was related to multiple physical function outcomes routinely used by clinicians. Post-ICU four-meter habitual gait speed was moderately correlated with FPN and DMN connectivity and strongly correlated with SMN connectivity. The patients in this study demonstrated slower gait speeds compared to normative age range data and prior post-ICU studies. Prior investigations in older adults, ages 70 to 80 years old, have demonstrated similar positive correlations of cortical network connectivity to gait speed [29]. Furthermore, connectivity with networks associated with attention and cognitive flexibility (i.e., FPN) and sensorimotor systems (i.e., SMN) demonstrated strong associations to composite scores of the SPPB. While mobility performance was grossly impaired, greater post-ICU connectivity appears to be tightly linked with physical function outcomes in patients recovering from ARF.

Physiologic variables associated with disease course and severity had a moderate association with network connectivity values. The majority of these relationships were found in the DMN. Furthermore, patients with longer hospital and ICU stays demonstrated higher DMN connectivity. Importantly, evidence has discerned the DMN to be one of the most metabolically active resting-rate networks in humans [30]. This may predispose this network to excessive vulnerability to ICU-related metabolic insults such as ischemia, systemic inflammation or toxicity, or disruption of blood-brain-barrier transportation. In this study, those with longer ventilation times, higher blood creatinine, and lower GFR demonstrated higher post-ICU DMN connectivity. Consequently, increased DMN connectivity could signify a compensatory response to accommodate for DMN network damage during ARF.

While the findings of the current study are based on small sample size, the moderate to strong relationships to clinical outcomes may warrant continued investigation. Future studies with larger sample sizes and across multiple recovery time points may be needed to determine the influence of an episode of ARF requiring ICU admission on functional connectivity and how CNS function influences outcomes. Additionally, this study did not control for ICU acquired delirium, which is associated with long-term cognitive impairments in critical illness survivors. Future studies should investigate or control for functional connectivity differences between patients with and without ICU-acquired delirium.

## Conclusions

The findings from our pilot study demonstrate that fMRI and objective neurocognitive testing is safe and feasible in the post-Medical ICU population. These exploratory findings suggest a critical illness may result in acute alterations to cortical networks underlying cognitive and motor function. The study provides preliminary evidence to support the use of connectivity analyses to assess underlying cortical mechanisms leading to deficits in multiple domains of health following ICU admission.
